# The effect of atorvastatin on inflammatory markers in sulfur mustard gas induced bronchitis: a randomized double-blinded, placebo-control clinical trial

**DOI:** 10.1186/s12890-021-01481-y

**Published:** 2021-04-01

**Authors:** Behrooz Momeni, Saeed Nazer, Seyed Masoom Masoompour, Bita Geramizadeh, Seyed Vahid Sajadi

**Affiliations:** 1 Department of Internal Medicine, School of Medicine, Shiraz University of Medical Sciences, Shiraz, 7193634154 Iran; 2 Student Research Committee, Shiraz University of Medical Sciences, Shiraz, Iran; 3 Non-Communicable Diseases Research Center, Department of Internal Medicine, School of Medicine, Shiraz University of Medical Sciences, Shiraz, Iran; 4 Transplant Research Center, Department of Pathology, School of Medicine, Shiraz University of Medical Sciences, Shiraz, Iran; 5 Department of Emergency Medicine, School of Medicine, Shiraz University of Medical Sciences, Shiraz, Iran

**Keywords:** Chronic bronchitis, Statins, Atorvastatin, Sulfur mustard gas

## Abstract

**Background:**

This study was performed to evaluate the anti-inflammatory effect of atorvastatin in patients with chronic bronchitis, exposed to sulfur mustard gas.

**Methods:**

In this randomized double-blinded clinical trial we recruited patients with chronic bronchitis after exposure to sulfur mustard gas. Ninety men 45–75 years old diagnosed with chronic bronchitis after exposure to mustard gas during the Iran-Iraq war, were randomly assigned to receive either atorvastatin (40 mg) or placebo once a day for 3 months. The interleukin 6 (IL-6), tumor necrosis factor α (TNF-α), procalcitonin, highly sensitive CRP and COPD assessment test (CAT) score was compared at baseline and after 12 weeks.

**Results:**

After consuming atorvastatin for 12 weeks, IL-6 level (mean difference [95%CI]; 0.2 [− 0.05, 0.5]), TNF-α (mean difference [95%CI]; − 0.07 [− 0.2, 0.07]), high sensitive CRP (mean difference [95%CI] − 0.1 [− 1.2, 0.9]), and procalcitonin (mean difference [95%CI]; 0.003 [− 0.02, 0.03]) did not change significantly. However, in the placebo group, only IL-6 (mean difference [95%CI]; 0.6 [0.2, 1.05]) decreased significantly after 12 weeks, but levels of high sensitive CRP (mean difference [95%CI]; − 0.3 [− 1.4, 0.8]) TNF-α (mean difference [95%CI]; − 0.2 [− 0.34, − 0.06]) and procalcitonin (mean difference [95%CI]; 0.02 [− 0.001, 0.04]) did not change significantly. After 12 weeks, the mean differences in TNF- α, IL-6 level, high sensitive CRP, procalcitonin, and CAT score did not significantly differ between the two groups.

**Conclusions:**

The administration of 40 mg atorvastatin for 3 months did not significantly change the inflammatory markers or the quality of life of patients exposed to mustard gas with chronic bronchitis.

*Trial registration*: IRCT, IRCT138904144312N1. Registered 16 August 2014, https://en.irct.ir/trial/4577.

**Supplementary Information:**

The online version contains supplementary material available at 10.1186/s12890-021-01481-y.

## Background

Sulfur mustard (2-bis-chloroethyl-sulfide) was discovered in 1821, and for the first time it was used during the First World War [1]. Due to its chemical alkylating compound, it can be easily absorbed through skin, respiratory system, ocular system, and genital tract [2]. Its toxicity is attributed to the lipophilic nature, which allows it to quickly penetrate target tissues and alkylate proteins, lipids and nucleic acids, resulting in DNA damage and cytotoxicity [3].

Unfortunately, sulfur mustard gas was used by the Iraqi army during the Iran–Iraq war 1980–1988. And a result, over 100,000 soldiers who did not have gas masks, suffered severe injuries, of which approximately 45,000 still continue to suffer the consequences of their exposure to this toxin [1]. Since the Iran–Iraq war, many Iranian war veterans have been admitted to hospitals with clinical manifestations of chemical gas poisoning, especially sulfur mustard gas [4]. These patients mostly suffer from respiratory problems, as the greatest causes of long-term disability, which include chronic bronchitis (58.9%) and asthma (10.6%) [5–7].

Both experimental and human trials have exhibited the involvement of inflammatory cells and mediators in sulfur mustard gas induced lung injuries. For instance, animal studies on rodents and pigs showed that exposure to sulfur mustard gas increases inflammatory cells in the upper and lower respiratory track for weeks or even months [8–13]. Furthermore, neutrophils and eosinophils numbers increased in the human lungs for long periods after exposure to sulfur mustard gas [14, 15]. However, the specific role of the inflammatory cells in sulfur mustard gas induced toxicity is not clear yet. In other models of lung injury, macrophages release inflammatory mediators with a key role in the pathogenesis of toxicity [16]; hence, it seems that they might play a similar role in the pulmonary response to sulfur mustard gas.

Hydroxymethyl-glutaryl (HMG) coenzyme A (CoA) reductase inhibitors (statins) have several modulatory effects, especially on neutrophils, which includes modulation of the innate and adaptive immune systems as well as the reduction of neutrophil migration [17–19]. This is in line with previous findings, stating that statins suppress major histocompatibility complex class II (MHC-II)-mediated T cell activation, in order to modulate host inflammatory cell recruitment by downregulating the activation of early inflammatory response gene nuclear factor B [20].

Therefore, we hypothesized that statins might be able to improve symptoms in patients with chronic bronchitis by reducing airway inflammation. Hence, the aim was to evaluate the anti-inflammatory effect of atorvastatin amongst patients with chronic bronchitis due to sulfur mustard gas inhalation. And the reason why we chose atorvastatin, was its low side effect and also because its anti-inflammatory effects are more than simvastatin [21].

## Methods

### Trial design

The present study is a two parallel randomized double-blinded, placebo-control trial group. We recruited only patients with chronic bronchitis due to sulfur mustard gas inhalation who referred to the pulmonary clinics affiliated with Foundation of Martyrs and Veterans Affairs, Shiraz, Iran. This study protocol was submitted and approved by institutional research board (IRB) of Ethics Committee of Shiraz University of Medical Sciences. After explaining the study objectives, all the participants gave their written informed consent. The trial was registered with the Iranian Clinical Trials Registry (IRCT138904144312N1; www.irct.ir).

### Participants

Ninety men between the age of 45–75 years old diagnosed with chronic bronchitis due to mustard gas inhalation during the eight years Iran-Iraq war were consecutively recruited from the outpatient clinic (chronic bronchitis characterized by a cough productive of sputum daily for over three months duration during two consecutive years and airflow obstruction). Exclusion criteria included a history of exacerbation of symptoms within the past 4 weeks, connective tissue disease, sarcoidosis, eosinophilic granuloma, pneumoconiosis, lymphoma, carcinomatous, active tuberculosis, chronic liver disease, and currently taking statins or those who used it within the last 3 months prior to the study. In addition, those who smoked or were former smokers, who had stopped smoking less than 1 year were also excluded from the study.

### Interventions and outcomes

The patients were randomly assigned to receive either atorvastatin (40 mg) or placebo (starch pills, made by Shiraz Pharmacology School, Iran), given orally once a day for 3 months. The shape and packing of both pills was similar, so patients and the researches were blinded to the treatment group allocation.

Demographic data; including age, body mass index (BMI), heart rate, respiratory rate, systolic and diastolic blood pressure, and drug history of each patient was recorded in a date sheet. In addition, blood tests including total cholesterol, triglyceride (TG), liver-function tests (LFTs), hemoglobin (Hb), and spirometry were recorded at the baseline. The primary outcome of this study was to assess systemic inflammation status at 3 months compared with baseline, measured by white blood cell (WBC) count, interleukin 6 level (IL-6), and tumor necrosis factor α (TNF-α). Also, we considered COPD assessment test (CAT) as the secondary outcomes. CAT is a patient-completed instrument to assess and quantify health-related quality of life and symptom burden in patients with COPD. It comprises of 8 questions, and each present a semantic 6-point (0–5) differential scale, providing a total score out of 40. The higher scores exhibit the severity of COPD impact on a patient’s life. [22].

### Measurements

All assessments were performed at baseline and the end of intervention. The IL-6 and TNF-α, concentrations were measured with enzyme linked immunosorbent assay (ELISA) commercial kits (Platinum, Austria) according to the manufacturer’s instructions. The reference range of IL-6 and TNF-α serum level were considered 1.8 pg/ml and up to 2.8 pg/ml. The high sensitive CRP concentration was measured with enzyme linked immunosorbent assay (ELISA) commercial kits (Diagnostics Biochem Canada Inc., Canada) according to the instructions of manufacturer. The serum level of high sensitive CRP less than 10 mg/l was considered as normal. The procalcitonin (PCT) level was measured via an automatic analyzer, the VIDAS® B.R.A.H.M.S PCT assay (bioMérieux, Marcy L'Etoile, France). The reference range of PCT was less than 150 pg/ml. BMI was calculated, using the weight and height measurements. Blood pressure was measured after a 5-min resting period with the individual sitting in a chair and determined, using a standard mercury sphygmomanometer. Moreover, the total CAT score was calculated for each patient by summing the points for each variable.

### Randomization

Randomization sequence was created, using random block sizes of 4 and 6. On the order of referral, the participants were allocated 1:1 into two groups. Study pills were allocated in separate packs blinded and labeled, using a four-digit code. The information regarding which codes correspond to what treatment was maintained secret by the project coordinator. The patients, attending physicians, staff involved in the pulmonary clinics, and members collecting and analyzing data were all blinded to the intervention allocation.

### Statistical analysis

All statistical analyses were performed with the Statistical Package for Social Sciences version 19.0 (SPSS Inc., Chicago, IL, USA). We estimated that a total of 90 participants required to detect significant difference between the groups, with a two-tailed α of 0.05 and a (1-β) of 0.80, for a comparison of 2 independent mean of outcome with effect size of 0.6. The Kolmogorov–Smirnov test was used to test normality of variables’ distribution. The baseline characteristics of both groups were compared, using X2 tests or Fisher’s exact test for proportions. For continuous variables, independent groups were compared, using the t-test or Mann–Whitney test, whereas paired comparison was made, using paired t-test or Wilcoxon test. Data are reported as means ± SD. A two-sided *P* value less than 0.05 was considered to be statistically significant. The effect size and 95% confidence interval of effect size of variables were calculated, using online calculators [23, 24].

## Results

**Fig. 1:**
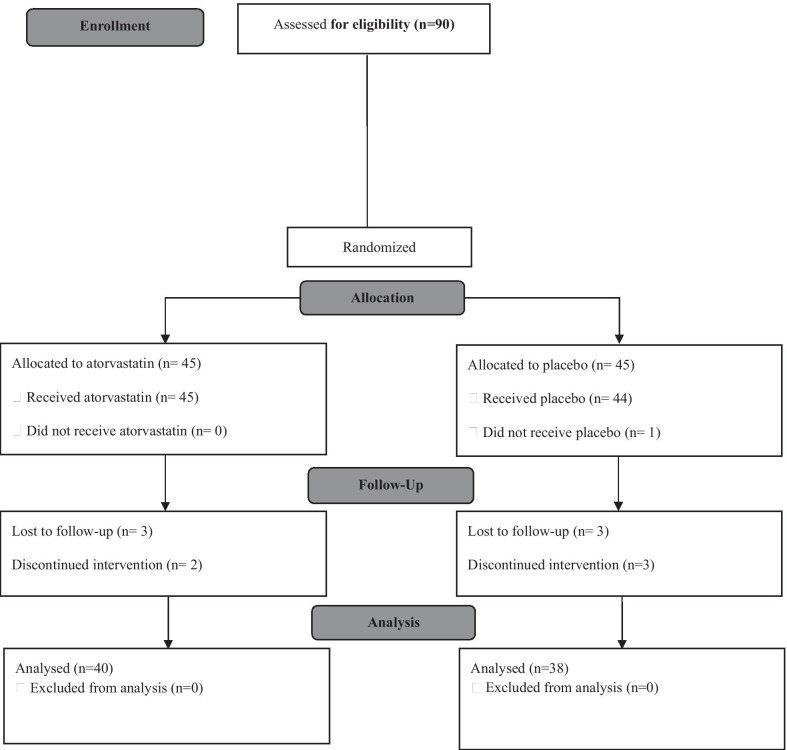
CONSORT 2010 study flow chart

Out of the 90 patients assessed for eligibility, one individual declined further participation. Thus, the final number of patients being randomized into two groups was 89 (45 in atorvastatin and 44 in the placebo groups). Three participants in the placebo group and two in the atorvastatin group left the study due to personal reasons and three patients from each group were lost to follow-up. Finally, 40 patients were enrolled in atorvastatin group and 38 were enrolled in the placebo group (Fig. 1). Three patients in each group were on long term oxygen therapy.

The mean age of the patients was 50.3 ± 5.7 (range 45–71) years and the mean BMI of the patients was 26.1 ± 5.1 kg/m^2^. The baseline characteristics of the patients are reported in four main categories including; demographics & clinical, baseline spirometry, blood tests, systemic inflammation markers, quality of life and medications (Table 1). Except for lower serum ALT (U/ml) in the atorvastatin group (mean difference [95% CI] − 8.1 [− 14.7, − 1.5]), there was no significant difference between the two study groups with respect to baseline characteristics. The mean CAT score was 29.4 ± 7, ranging from 13 to 40 in atorvastatin group and 28.1 ± 6, ranging from 14 to 40 in the placebo group (mean difference [95% CI]:0.57 [− 2.5, 3.7]).

**Table 1:** The baseline characteristics of the study patients

	Atorvastatin Group (n = 40)	Placebo Group (n = 38)	Mean difference (95% CI)	Effect size (95% CI)	*p* value
Demographics & clinical^a^					
Age (years)	51 ± 5.5	49.7 ± 5.9	1.4 (− 1, 3.8)	− 0.25 (− 0.7, 0.19)	0.10^c^
BMI (kg/m^2^)	27 ± 5.5	25 ± 4.3	1.8 (− .03, 3.9)	− 0.4 (− 0.85, 0.04)	0.08
Heart rate (beats/min)	78.6 ± 9	78.9 ± 10	− 0.3 (− 4.2, 3.7)	0.03 (− 0.41, 0.47)	0.84^c^
Respiratory rate (inhalation-exhalation cycles/min)	15.7 ± 2.3	15.1 ± 2.3	0.6 (− 0.4, 1.6)	− 0.26 (− 0.7, 0.18)	0.23^c^
Systolic blood pressure (mmHg)	115.6 ± 14	119.2 ± 14	− 3.6 (− 9.5, 2.4)	0.25 (− 0.18, 0.7)	0.26^c^
Diastolic blood pressure (mmHg)	72.9 ± 9	76.1 ± 8	− 3.2 (− 6.8, 0.3)	0.37 (− 0.07, 0.82)	0.07^c^
Mean FEV1/FVC	66.4 ± 9	66.7 ± 10	0.007 (− 0.035, 0.049)	0.03 (− 0.41, 0.47)	0.90
COPD classification					
FEV1 ≥ 80%, n (%)	22 (55%)	20 (54.1%)			0.97
50 ≤ FEV1 < 80, n (%)	11 (27.5%)	11 (29.7%)			
30 ≤ FEV1 < 50, n (%)	7 (17.5%)	6 (16.2%)			
Blood tests^a^					
Hemoglobin (g/dl)	15.4 ± 1.9	15.7 ± 1.6	− 0.3 (− 1, 0.5)	0.17 (− 0.27, 0.61)	0.46
Serum AST (U/ml)	21.8 ± 7	24.1 ± 12	− 2.3 (− 6.4, 1.8)	0.23 (− 0.21, 0.68)	0.49^c^
Serum ALT (U/ml)	20.6 ± 10	28.7 ± 19	− 8.1 (− 14.7, − 1.5)	0.53 (0.08, 0.98)	0.007^c^
Serum alkaline phosphatase (U/l)	206 ± 47	193 ± 49	12 (− 8, 32)	− 0.27 (− 0.71, 0.17)	0.23
Total Cholesterol (mg/dl)	206 ± 42	211 ± 85	− 4.7 (− 32, 23)	0.07 (− 0.36, 0.51)	0.74
Low-density lipoprotein (mg/dl)	124 ± 33	119 ± 30	4.8 (− 8.6, 18.3)	− 0.15 (− 0.6, 0.28)	0.47
TG (mg/dl)	174 ± 89	149 ± 114	25 (− 18, 67)	− 0.24 (− 0.69, 0.2)	0.25
Systemic inflammation markers^a^					
White blood cells (× 10^3^ cells per ml)	7.6 ± 2.5	6.7 ± 1.7	0.9 (− 0.09, 1.8)	− 0.41 (− 0.86,0.03)	0.052
IL-6 (pg/ml)	0.92 ± 0.87	1.1 ± 1.3	− 0.16 (− 0.67, 0.35)	0.16 (− 0.28, 0.6)	0.81^c^
TNF-α (pg/ml)	2.1 ± 0.4	1.9 ± 0.3	0.16 (− 0.01, 0.34)	− 0.4 (− 0.85, 0.04)	0.10^c^
HsCRP (ng/ml)	5.35 ± 3.2	4.10 ± 2.9	1.2 (− 1.2, 2.6)	− 0.4 (− 0.85, 0.04)	0.07
Procalcitonin (pg/ml)	0.043 ± 0.18	0.038 ± 0.07	0.005 (− 0.06, 0.07)	− 0.03 (− .048, 0.4)	0.20^c^
Quality of life^a^					
CAT score	29.4 ± 7	28.1 ± 6	1.24 (− 1.46, 3.95)	− 0.19 (− 0.64, 0.24)	0.36
Medication^b^					
Inhaled corticosteroids	4 (10%)	5 (14.7%)			0.81
Inhaled anticholinergics	6 (15%)	4 (11.1%)			0.77
Inhaled β2 agonists	19 (47.5%)	15 (41.7%)			0.72
Antihypertensive	13 (31.7%)	13 (38.2%)			0.67
Antidiabetics	5 (12.5%)	4 (11.8%)			0.87
Mucolytic	15 (37.5%)	14 (41.2%)			0.79

^a^Average, standard deviation

^b^Frequency and percentages

^c^Mann–Whitney test

There was no statistically significant difference between the treatment interval with respect to white blood cells (WBCs) in the atorvastatin group (mean difference [95% CI]: 0.01 [− 0.6, 0.63]), and likewise in the placebo group (mean difference [95% CI]: − 0.01 [− 0.45, 0.41]). In other words, in comparison with the placebo group, WBC did not significantly change in the atorvastatin group at the end of the study (mean difference [95% CI]: − 0.03 [− 0.8, 0.7]). Thereafter, the effects of atorvastatin and placebo on the serum levels of TNF-α, IL-6, high sensitive CRP, and procalcitonin were investigated after 12 weeks of treatments. After 12 weeks of using atorvastatin (n = 40), the level IL-6 (mean difference [95%CI]; 0.2 [− 0.05, 0.5]), TNF-α (mean difference [95%CI]; − 0.07 [− 0.2, 0.07]), high sensitive CRP (mean difference [95%CI] − 0.1 [− 1.2, 0.9]), and procalcitonin (mean difference [95%CI]; 0.003 [− 0.02, 0.03]) did not change significantly. In the placebo group (n = 38), only IL-6 (mean difference [95%CI]; 0.6 [0.2, 1.05]) significantly decreased after 12 weeks, but the levels of high sensitive CRP (mean difference [95%CI]; − 0.3 [− 1.4, 0.8]) TNF-α (mean difference [95%CI]; − 0.2 [− 0.34, − 0.06]) and procalcitonin (mean difference [95%CI]; 0.02 [− 0.001, 0.04]) did not change significantly. The mean differences in levels of TNF- α, IL-6, high sensitive CRP, and procalcitonin did not differ statistically between the study groups after 12 weeks. Table 2 shows changes in inflammatory markers after 12 weeks in the atorvastatin and placebo groups and between groups.

**Table 2:** Comparing outcomes within and between two study group

	Atorvastatin group (n = 40)				Placebo group (n = 38)				Difference between groups after 12 weeks		
	Baseline	12 weeks	Mean difference (95% CI)	Effect size (95% CI)	Baseline	12 weeks	Mean difference (95% CI)	Effect size (95% CI)	Mean difference (95% CI)	Effect size (95% CI)	*p* value
WBC (× 103 cells/ml)	7.6 ± 2.5	7.4 ± 1.8	0.01 (− 0.6, 0.63)	0.007 (− 0.42, 0.43)	6.7 ± 1.7	6.7 ± 1.5	− 0.01 (− 0.45, 0.41)	− 0.01 (− 0.35, 0.33)	− 0.03 (− 0.8, 0.7)	0.01 (− 0.42, 0.46)	0.93
IL-6 (pg/ml)	0.92 ± 0.87	0.69 ± 0.7	0.2 (− 0.05, 0.5)	0.15 (− 0.15, 0.46)	1.1 ± 1.3	0.46 ± 0.3	0.6 (0.2, 1.05)	0.64 (0.43, 0.86)	0.4 (− 0.09, 0.9)	− 0.36 (− 0.81, 0.08)	0.07
TNF-α (pg/ml)	2.1 ± 0.4	2.2 ± 0.3	− 0.07 (− 0.2, 0.07)	− 0.19 (− 0.27, − 0.11)	1.9 ± 0.3	2.2 ± 0.4	− 0.2 (− 0.34, − 0.06)	− 0.55 (− 0.63, − 0.47)	− 0.13 (− 0.3, 0.07)	0.29 (− 0.15, 0.74)	0.20
HsCRP (ng/ml)	5.35 ± 3.2	5.49 ± 3.4	− 0.1 (− 1.2, 0.9)	− 0.04 (− 0.76, 0.67)	4.10 ± 2.9	4.45 ± 3.2	− 0.3 (− 1.4, 0.8)	− 0.11 (− 0.78, 0.55)	− 0.2 (− 1.7, 1.3)	0.06 (− 0.38, 0.5)	0.78
Procalcitonin (pg/ml)	0.043 ± 0.18	0.04 ± 0.15	0.003 (− 0.02, 0.03)	0.01 (− 0.02, 0.05)	0.038 ± 0.07	0.02 ± 0.05	0.02 (− 0.001, 0.04)	0.3 (0.29, 0.32)	0.02 (− 0.01, 0.05)	− 0.22 (− 0.67, 0.21)	0.15
CAT score	29.4 ± 7	22 ± 5.6	7.8 (5.3, 10.3)	1.3 (− .005, 2.6)	28.1 ± 6	19.6 ± 7.2	8.4 (6.4, 10.4)	1.25 (− 0.26, 2.78)	0.57 (− 2.5, 3.7)	− 0.09 (− 0.53, 0.35	0.71
Cholestrol (mg/dl)	206 ± 42	160 ± 44	42 (29, 55)	1.03 (− 8.6, 10.6)	211 ± 85	180 ± 31	18 (9.3, 27)	0.56 (− 7, 8.1)	− 24 (− 39.5, − 8.7)	0.7 (0.24, 1.15)	0.003
Low-density lipoprotein (mg/dl)	124 ± 33	100 ± 37	22 (10, 33)	0.61 (− 6.8, 8.1)	119 ± 30	116 ± 24	3.6 (− 4.2, 11.5)	0.11 (− 5.7, 6.01)	− 18 (− 31.6, − 4.7)	0.58 (0.13, 1.04)	0.009

We compared changes of CAT score after the study interventions in each group. Although after 12 weeks in the atorvastatin and placebo groups the CAT score have met the minimum clinically important difference of a 2-unit reduction (mean difference [95% CI]: 7.8 [5.3, 10.3] and mean difference [95% CI]: 8.4 [6.4, 10.4] respectively), its mean difference did not change significantly between the two groups (mean difference [95% CI]: 0.57 [− 2.5, 3.7]) (Table 2).

## Discussion

This is the first study to evaluate the effect of atorvastatin on systemic inflammatory markers and quality of life in mustard gas induced bronchitis with a randomized, control and double-blinded designed.

The primary goal of this study was to evaluate the hypothesis that can atorvastatin reduce inflammatory markers in patients with mustard gas induced chronic bronchitis? However, this study could not find any significant reduction in the atorvastatin group inflammatory markers (IL6, TNF-α, HsCRP, and procalcitonin) at the level of 5%. Nevertheless, TNF-α serum levels increased significantly in the placebo group. Although the exact explanation is unclear, the IL6 reduction in this group may be explained by regression to mean theory [25].

In one hand, several studies could show the anti-inflammatory effect of statin [26, 27]; however, few could not [28, 29]. Considering the significant reduction in serum cholesterol level in the atorvastatin group, it is unlikely that poor adherence to the study protocol was the reason for the failure to reach statistical differences between groups. The lipid lowering effect of statins is independent of CRP lowering effect [26]. The benefit of intensive statin therapy is likely due to reduced level of both LDL and CRP. In contrary to Devaraj et al. [26] that had hypothesized the early benefit of statins might have been related to reduction of CRP presented preceding to their lipid lowering effect, our results showed lipid lowering effect without statistically significant reduction in HsCRP. The optimal level of statin to obtain the anti-inflammatory goals remains to be established; the anti-inflammatory effect of atorvastatin might require higher dose or longer duration. As it was stated in the method section, we included patients with stable mustard gas induced bronchitis, which might explain why the level of inflammatory markers was not statistically significant between the groups. On the other hand, the main site of inflammation in bronchitis is the lungs, and to be more precise, as Kaczmarek et al. [28] had suggested, it might be better to assess the inflammatory markers in bronchoalveolar washing.

We had initially thought that the power of our study was sufficient and acceptable to detect relatively moderate differences between atorvastatin and placebo groups; however, due to wider standard deviation than expected, it is likely that this study was slightly underpowered on post-hoc power analysis by G power [30].

The CAT score is a disease-specific instrument for assessing the severity of COPD [22, 31, 32]. Although the CAT score of our participants met the minimum of clinical important differences [33] of a 2-unit reduction in both groups, its mean differences was not significant between groups (mean difference [95% CI]: 0.57 [− 2.5, 3.7]). The CAT score reduction in the placebo group can be explained by placebo effect [34]. In contrary to our findings, Maneechotesuwan et al. [35] studied the effect of simvastatin 20 mg daily versus placebo on sputum inflammatory markers, airway inflammation, and CAT score of 30 patients with stable COPD, and found clinically significant important reduction in CAT score after statin administration. Mandal and colleagues, in a clinical trial assessing the role of atorvastatin in treating bronchiectasis, reported that the patients who received statins had a better quality of life (the St George's Respiratory Questionnaire) in comparison to those who had received placebo, nevertheless they did not reach minimum clinically important difference of a 4-unit reduction in SGRQ score [36].

### Limitations

This study had two limiting factors that debilitated the results. First, the participants were in relatively stable state of their disease, which might have reduced the effectiveness of statin. Second, we merely focused on few systemic inflammatory markers in a limited period of time, while the main source of inflammation in patients with bronchitis is the lung.

Therefore, larger prospective randomized controlled trials with longer follow up that focus on the respiratory tract indices, like exhaled air condensate or bronchoalveolar lavage, both during stable periods and exacerbations [28] and/or other systemic inflammatory, such as monocyte-macrophage function [26] is warranted.

## Conclusion

Despite its limitations, this study provides evidence that administration of 40 mg atorvastatin for 3 months cannot significantly reduce systemic inflammatory factors in the patients with chronic bronchitis due to sulfur mustard gas inhalation.

## Supplementary Information

**Additional file 1.** The raw data of this study.

## Data Availability

All data generated during this study are included in this manuscript (Additional file 1).

## Abbreviations

BMI: Body mass index
CAT: COPD assessment test score
CRP: C reactive protein
COPD: Chronic obstructive pulmonary disease
Hb: Hemoglobin
IL-6: Interleukin 6
LFTs: Liver-function tests
PCT: Procalcitonin
TG: Triglyceride
TNF-α: Tumor necrosis factor α
